# Serotonergic control of spine morphology during growth in zebrafish

**DOI:** 10.64898/2026.09.20.753056

**Published:** 2026-10-21

**Authors:** Stevana R. Schauer, Bryson Tyler B. Ricamona, Maximilian R. Segeberg, Laura Desban, Zane L. Walsh, Judith S. Eisen, Daniel T. Grimes

## Abstract

Scoliosis is a three-dimensional deformity of the vertebral column that commonly emerges during growth, yet the biological mechanisms that initiate and drive curve progression remain poorly understood. Serotonin (5-hydroxytryptamine [5-HT]) has long been linked to scoliosis through its relationship with pineal melatonin signaling and through genetic associations with human disease, but its role in spinal morphogenesis has remained unclear. Here, using zebrafish, we show that serotonergic signaling is required for normal spine morphology. Transient exposure to 5-HT during development induces later spinal abnormalities, while loss of Tph2, an enzyme required for neuronal 5-HT synthesis, causes progressive three-dimensional spinal curvature during juvenile growth. *tph2* mutants nevertheless exhibit initially normal vertebral patterning, arguing against an early skeletal defect as the initiating cause of curvature. Additionally, sustained ablation of *exorh* -positive pineal photoreceptors does not induce spinal curvature, arguing against a pineal origin for the phenotype. The Reissner fiber, which has also been linked to spinal curvature in zebrafish, formed normally in *tph2* mutants. Instead, mutants showed a marked reduction in Fev-expressing intraspinal serotonergic neurons together with increased locomotor activity, characterized by longer swim bouts and shorter periods of inactivity between bouts. Importantly, these neuronal and motor defects were evident before the onset of overt spinal curvature. Together, these findings identify neuronal serotonin as a critical regulator of spinal stability and support a model in which altered serotonergic control of motor function changes the mechanical environment experienced by the growing spine, increasing its susceptibility to progressive curvature.

## Full Text Availability

The license terms selected by the author(s) for this preprint version do not permit archiving in PMC. The full text is available from the preprint server.

